# Questions Frequently Asked of Healthcare Professionals: A 2-Year Data Survey Conducted at a Medical Center

**DOI:** 10.1155/2018/4895850

**Published:** 2018-02-14

**Authors:** Chi-Lien Hou, Ying-Hao Lu, Shun-Ching Chien, Hsu-Hui Chen, Chung-Yu Chen

**Affiliations:** ^1^Department of Pharmacy, Kaohsiung Medical University Hospital, Kaohsiung, Taiwan; ^2^Department of Emergency, Kaohsiung Medical University Hospital, Kaohsiung, Taiwan; ^3^Department of Information Technology, Kaohsiung Medical University Hospital, Kaohsiung, Taiwan; ^4^School of Pharmacy, Kaohsiung Medical University, Kaohsiung, Taiwan

## Abstract

In this descriptive, retrospective study, we analyzed the types of questions posed by healthcare professionals to drug counselors at a medical center and the types of provision of pharmaceutical advice solicited to improve pharmaceutical care quality and establish clear directions for clinical pharmacist training. We collected 8,558 questions posed by healthcare professionals (physicians, 38%; pharmacists, 39%; nurses, 23%) from the electronic drug information record system from May 2013 to April 2015 in one medical center. Overall, 52% and 45% of calls came from outpatient and inpatient departments, respectively. Telephone was the main route of provision of pharmaceutical advice (total, 6,035 questions; 72%), and hospital/electronic formulary was the main reference type (43%). The top 10 topics were dosage, alternatives, drug name, usage, adverse drug reactions, medication suggestion, drug compatibility, national health insurance criteria, mechanism, and indications. Pharmacological classification inquiries most frequently addressed antimicrobial agents (20%), and vancomycin was the top single drug. Finally, 67% of calls were completed in 5 minutes. Our results suggest that the systematic organization of issues into a searchable database would reduce inquiry durations and improve work efficiency. Furthermore, the availability of various search tools and methods would quickly provide healthcare professionals with provision of drug information needed to improve patient medication safety.

## 1. Introduction

In Taiwan, provision of pharmaceutical advice services is considered an important professional responsibility of pharmacists. The amended Taiwan Pharmacists Act of 2007 expressly stipulates that pharmacists shall conduct functions related to pharmaceutical care [1], and the Pharmacists Act Implementation Regulations stipulates that pharmaceutical care must include provision of pharmaceutical advice [2]. In addition, the 2016 Taiwan Hospital Assessment Benchmark and Assessment Program stipulates that it is necessary to provide “immediate and correct provision of drug information services for medical professionals and records” [3].

Clients seeking provision of pharmaceutical advice include both patients and healthcare professionals. According to previous studies, patients usually inquire about drug usage, because they do not understand how to use their prescribed drugs [4, 5]. However, healthcare professionals face very different challenges, as they must address patients' complex conditions in emergency or diverse clinical situations. Accordingly, healthcare professionals expect pharmacists to provide correct responses to their inquiries in the shortest possible time. Therefore, pharmacists who understand the needs of healthcare professionals in terms of drug information and frequently asked questions can search for and provide correct drug information in a relatively efficient manner.

Most studies of provision of pharmaceutical advice have analyzed the types of services provided to patients or have presented an overall discussion of the services provided to both patients and healthcare professionals [6–9] but have rarely addressed the specific needs of healthcare professionals. Therefore, in the present study, we aimed to analyze the types of questions posed by healthcare professionals at a medical center, explore the needs of different types of professionals (e.g., doctors, pharmacists, and nurses), and determine the type of drug information that would best meet the needs of clinicians providing care while enhancing medication safety among patients.

## 2. Methods

For this retrospective, cross-sectional study, we collected electronic drug information records entered into a medical center database from May 2013 to April 2015 (i.e., 2-year study period). Excel software (Microsoft Corp., Redmond, WA, USA) was used to collect and analyze data concerning the drug-related information solicited by healthcare professionals (i.e., physicians, pharmacists, and nurses). The proportions of provision of pharmaceutical advice were analyzed with respect to the following categories: physicians, pharmacists, and nurses (number of consultations per category/total number of healthcare professional consultations); consulting approach (number of consultations per type/total number of healthcare professional consultations); and inquiry unit (all types of inquiry units/total number of healthcare professional consultations). The top 10 reference sources, top 10 pharmacological categories, top 20 drugs, top 10 question categories, reply time length, and inquiry unit were also further divided into outpatient and hospitalized categories, which were compared to identify differences. This study was approved by the Institutional Review Board of Kaohsiung Medical University Hospital (KMUHIRB-E(I)-20160175).

## 3. Results

During the 2-year study period, a total of 20,009 cases of provision of pharmaceutical advice were registered in the electronic record system; of these, 8,558 (43%) involved consultations with healthcare professionals. Physicians, pharmacists, and nurses were responsible for 38% (3,277), 39% (3,367), and 23% (1,914 times) of these cases, respectively. Furthermore, 52% (4,466), 45% (3,797), 1% (108), and 2% (187) of inquiries were attributed to the outpatient, inpatient, emergency, and other departments, respectively. Further classification of healthcare professionals revealed that 61% and 36% of physicians, 29% and 67% of pharmacists, and 42% and 55% of nurses in inpatient and outpatient departments, respectively, made inquiries. In other words, outpatient pharmacists and nurses and hospital physicians were more likely to initiate a consultation.

Regarding the top 10 issues raised, doses, alternative medicines, drug names, and usage accounted for 62% of all inquiries (Table 1). By professional category, physicians mainly asked about doses, alternative medicines, and drug names (Table 2); nurses asked about alternative drugs, drug names, and intravenous (IV) compatibility (Table 3); and pharmacists asked about doses, usage, and national health insurance (Table 4). These patterns of inquiry also differ within the professional type categories. For example, hospital physicians more frequently inquired about common issues related to doses, adverse drug reactions, and medication recommendations, whereas outpatient physicians most frequently asked about alternative medicines, drug names, and doses (Table 2). Inpatient nurses often asked about IV compatibility, drug stability, and usage problems, whereas outpatient nurses asked about alternative drugs, drug names, and usage problems (Table 3). Outpatient pharmacists tended to ask about doses, usage, and national health insurance; ward pharmacists similarly asked about doses and usage but also inquired about medication advice (Table 4).

**Figure 1:**
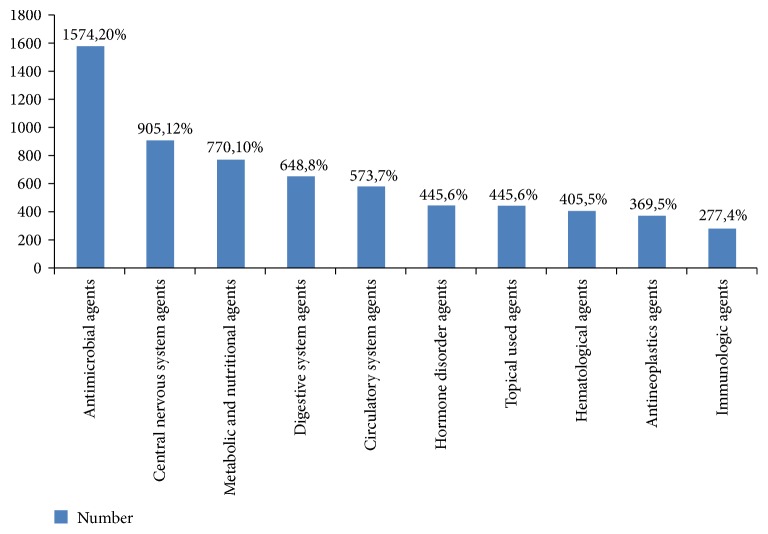
Top 10: drug pharmacological classification of frequently asked questions of healthcare professionals.

Regarding pharmacological classifications, the inquiries most frequently addressed antimicrobial drugs (20%), followed by central nervous system drugs (12%) and metabolic drugs (10%), as shown in Figure 1. As shown in Table 5, the top 20 drugs included seven antibiotic agents, with vancomycin and potassium chloride in the first and second positions, respectively. Similarly, residents most frequently inquired about antibiotics, which comprised the top nine drugs in this category (Table 6).

**Figure 2:**
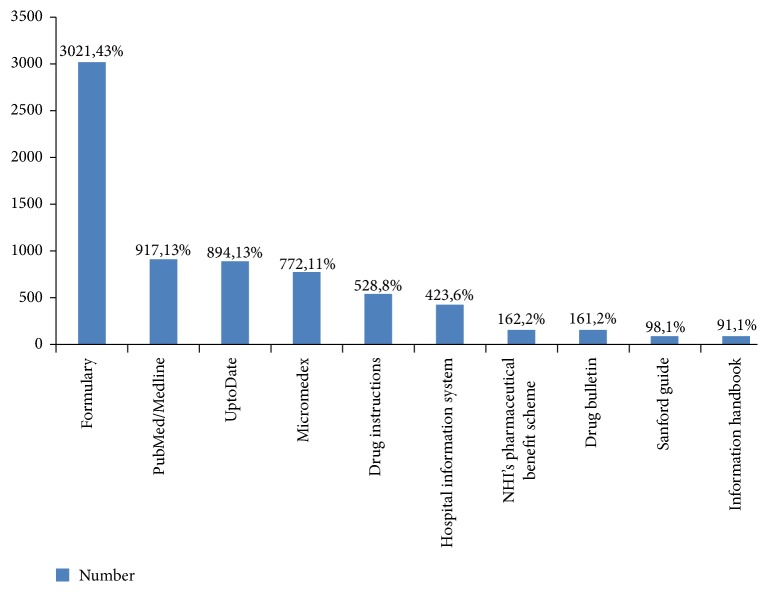
Top 10: reference sources for provision of pharmaceutical advice.

Most consultations were made via telephone (72%), followed by over-the-counter consultations (17%) and clinical visits (11%). Regarding the sources of reference material, pharmacists most often reported consulting the hospital formulary (43%), followed by PubMed/Medline (13%), UpToDate® (13%), Micromedex (11%), and other databases (Figure 2). Most inquiries (67%) were completed within 5 minutes, followed by 5–10 (21%), 10–30 (8%), 30–60 (1%), and >60 minutes (3%).

## 4. Discussion

In this retrospective study of drug-related inquiries in a hospital database, we found that pharmacists and physicians made 39% and 38% of inquiries, respectively. In other words, pharmacists made as many inquiries as physicians did, a finding that can likely be attributed to the need for pharmacists to evaluate a large number of prescriptions every day. Accordingly, pharmacists would be more likely to encounter dubious or unclear prescriptions.

We further noted that more inquiries originated in the outpatient department relative to the hospital and emergency departments, likely because the former department encounters more patient issues and handles the lowest number of emergency cases. Among physicians, most provisions of pharmaceutical advice were initiated by those working in the hospital. This can be attributed to the more serious clinical symptoms and situations of inpatients, who often require a variety of drugs. Therefore, inpatient physicians must monitor the risks of drug interactions or drug-induced side effects and, in some cases, the provision of pharmaceutical advice to ensure the effectiveness of treatment and to avoid side effects. In contrast, outpatients tend to have more stable conditions, and outpatient physicians tend to work in specialist clinics with relatively narrow ranges of medication and thus have more experience with the use of those drugs. Accordingly, outpatient physicians would have a reduced need for provision of pharmaceutical advice. In contrast, we found that two-thirds of inquiries by pharmacists were made by outpatient professionals, possibly because more prescriptions are filled on an outpatient basis and outpatient divisions clearly; pharmacists can more easily find the problems of the prescription by division and diagnosis and then ask the consulting pharmacists. Outpatient nurses were also more likely to seek the provision of pharmaceutical advice relative to hospital nurses, although this might be related to the provision of assistance to outpatient care physicians who also tended to make more inquiries relative to their hospital counterparts.

In a previous review of drug counseling for patients and healthcare professionals, 475 of them were healthcare professionals. The common types of problems were indications, contraindications, interactions, and adverse reactions [10], and in our study healthcare professionals most frequently inquired about doses, followed by alternative drugs, drug name, usage, adverse drug reactions, medicine recommendations, IV compatibility, national health insurance, pharmacology/mechanism, and indications. Our results are roughly the same as the previous study [10]. This study's further analysis revealed that hospital physicians most frequently inquired about doses, adverse drug reactions, medication recommendations, drug compatibility, usage, and drug concentration. In this group, antibiotics represented the top nine drug inquiries, possibly because of the increased frequency of patients treated with antibiotics. Accordingly, physicians would solicit advice from pharmacists regarding doses, blood concentration monitoring, or dose adjustments for special patient groups. Hospital physicians also frequently asked about adverse drug reactions and medication recommendations, because hospitalized patients tended to exhibit severe clinical symptoms and more frequently developed multiple complications requiring multiple drugs. Given the risk of adverse drug reactions, physicians whose patients use multiple drugs would require assistance from pharmacists to clarify the symptoms caused by drugs.

Outpatient physicians most frequently asked about alternative options and drug names but less frequently about doses. The latter is likely attributable to the considerable experience of outpatient physicians with specialized drug treatment. The former likely results from the recent governmental requirement for pharmaceutical companies to seek Pharmaceutical Inspection Co-operation Scheme Good Manufacturing Practice certification. Some manufacturers could not meet the commission and were thus required to stop manufacturing or producing the drugs [11] and changes in the supply together with health insurance drug price surveys, hospital drug prices, and other factors led to an increase in the frequency of drug dressing. Accordingly, clinicians must often ask about alternative disease treatments or drug names. Furthermore, outpatient physicians are more willing than their hospital counterparts to try new drugs and tend to inquire whether the hospital has or has not adopted a new drug; therefore, pharmacists must learn to absorb information about new drugs to meet clinical needs. In contrast, hospital physicians made fewer inquiries about alternative drugs and drug names, possibly because the hospital tended to administer the original medications prescribed in outpatient and emergency settings. Additionally, the outpatient drug search system included both the trade and generic names, and an outpatient physician searching for the former may not be able to identify a replacement drug, leading to an inquiry. An in-hospital search, in contrast, would involve the generic name and would be less affected.

We observed that outpatient and hospital nurses made very different types of inquiries. The former tended to ask about alternative drugs, drug names, and usage, whereas the latter more frequently asked about drug compatibility, drug stability, and usage. We note that hospital inpatients are more frequently prescribed injection drugs. Therefore, ward nurses must understand concepts related to drug dilution, dilution stability, administration route, storage, and compatibility. In contrast, outpatients tend to receive oral drugs. However, as mentioned in the Results, outpatient nurses often assist physicians with inquiries about drug problems, leading to similar results for these two categories of outpatient professionals. Notably, outpatient physicians and nurses most often asked about alternative drugs. If a hospital cannot acquire the same type of drug in a timely manner, pharmacists must identify full or partial replacements and provide related advice to physicians. Whereas a computer system can prompt medical staff regarding the new drug name for a full replacement, pharmacists must remind medical staff of the usage and precautions of partial replacements.

Among pharmacists, both outpatient and inpatient professionals mostly inquired about drug doses. These professionals would be aware of abnormal prescribed doses or the need for dose adjustments among particular ethnic groups. Pharmacists also asked about drug usage and compliance with national health insurance.

Regarding pharmacological classification, antimicrobial agents were the most frequent topic of inquiry among hospital physicians, particularly with regard to injection dosage. The clinical use of antibiotics is complex, and advice from pharmacists is needed with regard to many issues, such as drug selection, dosage recommendations, alternative treatments, hepatic and renal dysfunction, dose adjustment for special populations, drug concentration monitoring time, dose adjustment monitoring, treatment recommendations, and compliance with national health insurance. Notably, another study of medical center statistics has found that antibiotics accounted for seven of the top 20 drugs associated with inquiries [6]. In the present study, vancomycin was the most frequently queried antibiotic. A pharmacist with an in-depth understanding of antibiotic therapy and familiarity with drug concentration monitoring and dose adjustment will be very helpful in solving clinical problems. Potassium chloride was the second most commonly used hospital drug, and nurses tended to ask about its compatibility with other injectable drugs. Similarly, many inquiries addressed compatibility issues related to antibiotics and IV nutritional preparations. In this regard, pharmacists in the present study most commonly sought information from drug compatibility references, as well as the literature and the Micromedex database.

Regarding advisory service quality, telephone consultation was the most frequently used and most convenient method of inquiry used by medical personnel in the present study. To provide information, pharmacists most frequently used the hospital/online formulary, which contained the dose, side effects, precautions, storage conditions, stability, pregnancy/breastfeeding indications, renal function adjustment information, price, health insurance status, drug supply status, appearance, food interaction, instructions, and other diversified information for each type of drug. Online pharmacopoeia, which provides the latest drug information, rapid query speeds, and simple methodology, was used very frequently. Medical center pharmacists also referred to secondary and tertiary literature databases, such as PubMed/Medline, UpToDate, and Micromedex.

Regarding response times, we found that two-thirds of the questions were answered within 5 minutes and that nearly 90% were addressed within 10 minutes. The remaining questions, however, addressed issues such as treatments for rare diseases, therapeutic dose adjustments for special groups, or alternative therapies and therefore required additional time. Medical staff prefer to receive answers to their inquiries as soon as possible. Therefore, to reduce the query times and provide more efficient answers, pharmacists must understand the context of the problem and the existing resources. When using secondary or tertiary literature sources, such as Micromedex, books, or Medline and other databases, however, some information may be difficult to obtain and consultations with experts or pharmaceutical companies may be required.

In addition to the above-mentioned issues regarding pharmacists' familiarity with query tools, the drug-related questions frequently posed by medical staff can be compiled into queryable formats, such as IV drug preparations, food–drug interactions, and Chinese herbal medicine interactions, which would save valuable time for pharmacists. A physician prescription system could be directly linked with the hospital prescription system and could thus query drug indications, doses, usage, side effects, drug appearances, and drug change information, as well as drug replacements, renal function adjustment doses, drug interactions, and national health insurance conditions as prompted by the computer system. Hospital physicians also frequently ask for vancomycin dose adjustments, which could be calculated conveniently by a program, rather than requiring a reply from a pharmacist; similarly, nurses often ask about drug stability and storage, compatibility, milling/cutting, and other information that could be displayed in the nursing computer system. Simultaneously, a drug information inquiry system that includes drug appearances, dosage recommendations, imitations, drug changes, drug supply statuses, and links to Micromedex and UpToDate could be established for medical personnel. In addition, more specific issues could be submitted for case discussions and shared with peers, the pharmacy department can regularly conduct pharmacological training for pharmacists [12], and medical personnel medication consultation records could be subjected to statistical analyses, with important issues announced via hospital news outlets. Pharmacists can participate in clinical rounds and case discussions, learning clinical knowledge and judgment laboratory data, and according to the patient's pathophysiological conditions, given the appropriate dose, dosage form recommendations [10, 13].

Provision of pharmaceutical advice is an important component of pharmaceutical-based care, and pharmacists are responsible not only for meeting legal and hospital requirements but also for the safety of the public. Pharmacists must, therefore, keep up with ever-evolving drug changes. The present study is expected to provide hospital pharmacists with support and indications for the cultivation of professional self-training and other training abilities in accordance with the convenience of a computerized system. These advances will improve the quality of drug care and ensure the safety of patients.

## Figures and Tables

**Table 1:** Top 10: type of questions from healthcare professionals.

	Type of questions	Number	%
(1)	Dosage	2618	22%
(2)	Alternative medicine	1809	15%
(3)	Drug name	1531	13%
(4)	Usage	1415	12%
(5)	Adverse drug reactions	961	8%
(6)	Recommendation of medicines	950	8%
(7)	IV compatibility	919	8%
(8)	NHI's criteria^*∗*^	724	6%
(9)	Pharmacology/mechanism	475	4%
(10)	Indication	448	4%

^*∗*^NHI: national health insurance.

**Table 2:** Top 10: type of questions from physicians, divided into OPD and IPD.

All physicians			Physicians in OPD^*∗*^			Physicians in IPD^*∗∗*^		
	Type of question	Number (%)		Type of question	Number (%)		Type of question	Number (%)
(1)	Dosage	710 (20%)	(1)	Alternative medicine	390 (32%)	(1)	Dosage	583 (27%)
(2)	Alternative medicine	466 (13%)	(2)	Drug name	266 (22%)	(2)	Adverse drug reactions	252 (12%)
(3)	Drug name	348 (10%)	(3)	Dosage	98 (8%)	(3)	Recommendation of medicines	211 (10%)
(4)	Adverse drug reactions	300 (9%)	(4)	NHI's criteria	72 (6%)	(4)	IV compatibility	146 (7%)
(5)	Recommendation of medicines	255 (7%)	(5)	Usage	56 (5%)	(5)	Usage	94 (4%)
(6)	IV compatibility	161 (5%)	(6)	Adverse drug reactions	41 (3%)	(6)	Therapeutic drug monitoring	83 (4%)
(7)	Usage	156 (4%)	(7)	Pregnancy	39 (3%)	(7)	Drug name	75 (3%)
(8)	NHI's criteria^*∗∗∗*^	113 (3%)	(8)	Recommendation of medicines	38 (3%)	(8)	Alternative medicine	69 (3%)
(9)	Drug interaction	103 (3%)	(9)	Drug interaction	36 (3%)	(9)	Drug interaction	64 (3%)
(10)	Therapeutic drug monitoring	84 (2%)	(10)	Lactating	21 (2%)	(10)	Medication history assessment	58 (3%)

^*∗*^OPD: outpatient department; ^*∗∗*^IPD: inpatient department; ^*∗∗∗*^NHI: national health insurance.

**Table 3:** Top 10: type of questions from nurses.

All nurses			Nurses in OPD			Nurses in IPD		
	Type of question	Number (%)		Type of question	Number (%)		Type of question	Number (%)
(1)	Alternative medicine	336 (17%)	(1)	Alternative medicine	293 (27%)	(1)	IV compatibility	197 (22%)
(2)	Drug name	316 (16%)	(2)	Drug name	272 (25%)	(2)	Drug stability	104 (12%)
(3)	IV compatibility	238 (12%)	(3)	Usage	76 (7%)	(3)	Usage	86 (10%)
(4)	Usage	165 (8%)	(4)	IV compatibility	36 (3%)	(4)	Storage	55 (6%)
(5)	Drug stability	130 (7%)	(5)	NHI's criteria	34 (3%)	(5)	Dosage	53 (6%)
(6)	Dosage	80 (4%)	(6)	Drug appearance	31 (3%)	(6)	Alternative medicine	37 (4%)
(7)	Storage	76 (4%)	(7)	Dosage	24 (2%)	(7)	Drug name	35 (4%)
(8)	Adverse drug reactions	51 (3%)	(8)	Adverse drug reactions	22 (2%)	(8)	Administration	33 (4%)
(9)	NHI's criteria	48 (2%)	(9)	Drug stability	21 (2%)	(9)	Recommendation of medicines	31 (4%)
(10)	Recommendation of medicines	44 (2%)	(10)	Storage	21 (2%)	(10)	Drug interaction	31 (4%)

**Table 4:** Top 10: type of questions from pharmacists.

All pharmacists			Pharmacists in OPD			Pharmacists in IPD		
	Type of question	Number (%)		Type of question	Number (%)		Type of question	Number (%)
(1)	Dosage	545 (18%)	(1)	Dosage	321 (17%)	(1)	Dosage	204 (21%)
(2)	Usage	398 (13%)	(2)	Usage	292 (15%)	(2)	Recommendation of medicines	110 (11%)
(3)	NHI's criteria	214 (7%)	(3)	NHI's criteria	155 (8%)	(3)	Usage	92 (9%)
(4)	Recommendation of medicines	182 (6%)	(4)	Alternative medicine	89 (5%)	(4)	Pharmacology/mechanism	92 (9%)
(5)	Pharmacology/mechanism	173 (6%)	(5)	Indication	87 (4%)	(5)	Adverse drug reactions	59 (6%)
(6)	Adverse drug reactions	136 (4%)	(6)	Pharmacology/mechanism	79 (4%)	(6)	Indication	39 (4%)
(7)	Indication	131 (4%)	(7)	Drug appearance	75 (4%)	(7)	Drug name	39 (4%)
(8)	Drug name	111 (4%)	(8)	Adverse drug reactions	74 (4%)	(8)	IV compatibility	35 (4%)
(9)	Alternative medicine	110 (4%)	(9)	Drug name	69 (4%)	(9)	NHI's criteria	34 (3%)
(10)	Drug appearance	82 (3%)	(10)	Recommendation of medicines	67 (3%)	(10)	Storage	34 (3%)

**Table 5:** Top 20: drugs of frequently asked questions of healthcare professionals.

	Drug	Number^*∗*^
(1)	Vancomycin	107
(2)	Potassium chloride 15%	76
(3)	Erythromycin eye ointment	69
(4)	Meropenem	65
(5)	Colimycin	58
(6)	Teicoplanin	57
(7)	Piperacillin + tazobactam	50
(8)	Tetracycline eye ointment	45
(9)	Pregabalin	44
(10)	Potassium gluconate	42
(11)	Imipenem + cilastatin	42
(12)	Fluconazole	41
(13)	Loratadine + pseudoephedrine	40
(14)	Clopidogrel	38
(15)	Oliclinomel N4-550E	38
(16)	Aspirin	37
(17)	Esomeprazole	37
(18)	Piracetam	36
(19)	Bacitracin/neomycin ointment	36
(20)	Warfarin sodium	35

^*∗*^Total number 4742.

**Table 6:** Top 10: drugs of questions from physicians in inpatient department.

	Drug	Number^*∗*^
(1)	Vancomycin	79
(2)	Teicoplanin	44
(3)	Colimycin	40
(4)	Fluconazole	34
(5)	Meropenem	33
(6)	Piperacillin + tazobactam	29
(7)	Imipenem + cilastatin	29
(8)	Tigecycline	20
(9)	Anidulafungin	19
(10)	Sodium valproate	16

^*∗*^Total  number 2149.

## References

[1] Department of Health, Execcutive Yuan, Pharamcist Act, 2007, http://law.moj.gov.tw/Eng/LawClass/LawAll.aspx?PCode=L0030066

[2] Department of Health (2009). *Executive Yuan. Pharmacists Act Implementation Regulations*.

[3] Joint Commission of Taiwan (2016). *Hospital Evaluation Benchmarks and Assessment Items*.

[4] Pang H. L., Lai C. C., Lei C. P., Kao H. C., Chien I. W., Hsu Y. H. Analysis on the effect of drug consultation service in a medical center.

[5] Yu C. I., Yu M. H. Analysis of drug consultation in psychiatric hospital.

[6] Wang Y. H. Analysis and application hospital common problem of drug counseling-to a medical center as an example.

[7] Huang H. I., Kuo M. H. Analysis and review of drug advisory service in a regional teaching hospitals.

[8] Chen Y. L., Lu Y. C., Yang Y. P. Analysis of drug consultation in a regional teaching hospital.

[9] Haugbílle L. S., Westh Sírensen E., Gundersen B., Holme Petersen K., Lorentzen L. (2002). Basing pharmacy counselling on the perspective of the angina pectoris patient. *Pharmacy world and science*.

[10] Zhu B. (2005). Review and Analysis the 2041 Cases of Drug Consultation in Our Hospital. *Journal of Modern Food and Pharmaceuticals*.

[11] Chen Y. H., Huang C. L., Li M. H., Chen P. Y., Chen H. F. (2010). Taiwan pharmaceutical companies to implement PIC/S GMP investigation and study , Annual Report of Food and Drug Research. *Taiwan pharmaceutical companies to implement PIC/S GMP investigation and study , Annual Report of Food and Drug Research*.

[12] Shao H., Li W. L., Chen G. M., Xu R. N., Zheng X. Y. (2005). Analysis and Countermeasures of Outpatient Drug Consultation Services , Pharmaceutical Care and Research. *Analysis and Countermeasures of Outpatient Drug Consultation Services , Pharmaceutical Care and Research*.

[13] Wu S. H., Hsu Y. T., Tseng W. L. (2013). Evaluation of the Efficacy of Pharmacist Consultation Program for Patients in an Elder Care Center. *Formosan Journal of Medicine*.

